# Effect of Fascial Distortion Model Treatment on Pain Intensity for Musculoskeletal Pain: A Systematic Review and Meta-Analysis

**DOI:** 10.7759/cureus.112144

**Published:** 2026-07-06

**Authors:** Alexander Ponce, Camille M Reaux, Kevin Lebo, Katlyn Joubert, Abigail Bryant, Alexander King, Kristie Petree

**Affiliations:** 1 Osteopathic Medicine, William Carey University, Hattiesburg, USA; 2 Neuromusculoskeletal Medicine, Virtua Medical Group, Sewell, USA

**Keywords:** fascia, fascial distortion model, fdm, manual medicine, musculoskeletal pain, omm, omt, osteopathic medicine, pain medicine, rehabilitation

## Abstract

Musculoskeletal pain is a common cause of disability and healthcare utilization worldwide. Manual therapies are frequently incorporated into multidisciplinary treatment strategies, and the Fascial Distortion Model (FDM) has been investigated as a treatment for a variety of musculoskeletal conditions. However, the effects of FDM on patient-reported pain have not previously been quantitatively synthesized. However, published evidence syntheses of FDM are limited in scope and have not comprehensively evaluated patient-reported pain outcomes across musculoskeletal conditions. The purpose of this systematic review and meta-analysis was to evaluate the comparative effectiveness of FDM versus alternative interventions on patient-reported pain outcomes and, secondarily, to evaluate pre-post changes in pain following FDM treatment. A systematic literature search of PubMed/MEDLINE, Google Scholar, the Cochrane Library, Semantic Scholar, and ClinicalTrials.gov was conducted from database inception through June 2026. The primary analysis compared post-intervention pain outcomes between FDM and non-FDM interventions in randomized controlled trials, while a secondary pre-post meta-analysis evaluated pain reduction following FDM treatment. Risk of bias was assessed using the Cochrane Risk of Bias 2 (RoB 2) and Risk Of Bias In Non-randomized Studies of Interventions (ROBINS-I) tools, and certainty of evidence was evaluated using the Grading of Recommendations Assessment, Development, and Evaluation (GRADE) framework. Fourteen studies involving 644 participants met the inclusion criteria. Compared with alternative interventions, FDM demonstrated a statistically significant reduction in pain (MD: -0.92; 95% CI: -1.70 to -0.14); however, the magnitude of benefit did not reach a clinically meaningful threshold. Across all included studies, FDM was associated with clinically meaningful reductions in pain from baseline (MD: -3.42; 95% CI: -4.23 to -2.61), although substantial heterogeneity was observed. Prespecified subgroup analyses demonstrated pooled mean differences of -4.14 (95% CI: -5.86 to -2.42) among studies utilizing diagnosis-specific FDM treatment approaches and -2.94 (95% CI: -3.73 to -2.16) among randomized controlled trials. According to the GRADE assessment, randomized controlled trials provided moderate-certainty evidence supporting an association between FDM and reduced pain. Overall, current evidence suggests that FDM is associated with reductions in musculoskeletal pain but does not appear to provide a clinically meaningful advantage over other conservative treatment approaches. Additional adequately powered randomized controlled trials are needed to better define the comparative effectiveness of FDM across musculoskeletal conditions.

## Introduction and background

Musculoskeletal disease is a leading cause of disability and morbidity worldwide, affecting an estimated 1.7 billion people according to the World Health Organization [1,2]. As populations continue to age, the prevalence and healthcare burden of musculoskeletal disorders are expected to increase. Because musculoskeletal disorders encompass a broad spectrum of conditions with varying degrees of pain severity, standardized assessment of pain is essential for evaluating treatment response. Patient-reported pain is most commonly quantified using validated instruments such as the Visual Analog Scale (VAS) and the Numerical Pain Rating Scale (NPRS). The VAS has demonstrated strong reliability and validity for assessing pain intensity, while the NPRS has been associated with high patient compliance and ease of administration [3,4]. These instruments provide standardized measures that enable the objective comparison of pain outcomes across clinical practice and research studies. The minimal clinically important difference (MCID) is generally considered to be approximately 2 points or 30% on a 0-10 scale [3]. Accordingly, these validated outcome measures provide a standardized framework for evaluating the effectiveness of pharmacologic and nonpharmacologic treatment strategies for musculoskeletal pain.

Musculoskeletal pain often results in substantial disability and healthcare utilization, necessitating a comprehensive, multimodal approach to management [5]. Current clinical guidelines from the American College of Physicians (ACP) recommend individualized treatment strategies that address both the physiologic and biopsychosocial contributors to pain through a combination of pharmacologic and nonpharmacologic interventions [6]. Pharmacologic management commonly includes non-opioid analgesics, adjuvant medications, and judicious use of opioid analgesics when clinically indicated [5,7,8]. Nonpharmacologic management encompasses a broad range of conservative therapies, including superficial heat, cryotherapy, transcutaneous electrical nerve stimulation (TENS), low-level laser therapy, cognitive behavioral therapy, and manual treatment techniques, each supported by varying degrees of clinical evidence [5,6]. For patients with persistent or refractory symptoms, pain intervention therapies may provide additional treatment options through minimally invasive procedures, including trigger point injections, facet and sacroiliac joint injections, epidural steroid injections, peripheral nerve blocks, and spinal cord stimulation [5]. Among conservative nonpharmacologic treatment approaches, manual therapies are commonly incorporated into the management of musculoskeletal disorders with the goal of reducing pain and improving function.

Manual therapy represents one category of conservative nonpharmacologic treatment for musculoskeletal pain and encompasses a variety of clinician-directed, hands-on interventions intended to reduce pain and improve physical function [9]. Common approaches include physical therapy, massage therapy, and osteopathic manipulative treatment (OMT) [10,11]. Systematic reviews have demonstrated that physical therapy, massage therapy, and OMT are associated with improvements in pain and functional outcomes across a variety of musculoskeletal conditions, although treatment effects vary by intervention and clinical diagnosis [11-14]. While these approaches differ in their specific techniques, many manual therapies target distinct anatomical structures, including joints, muscles, connective tissues, and fascia, with the goal of restoring function and alleviating pain [9,10]. Increasing understanding of the biomechanical and sensory roles of fascia has contributed to the development of manual therapies that specifically target fascial tissues [15]. Superficial and deep investing fascia are densely innervated with nociceptors. When mechanically or chemically stimulated, these nociceptors activate central nervous system pathways that precipitate the perception of pain [16,17]. 

The Fascial Distortion Model (FDM) is a fascial-based manual treatment approach developed by Stephen Typaldos, DO, for the management of musculoskeletal pain [17,18]. FDM conceptualizes some forms of musculoskeletal pain as arising from distortions within fascial tissues that are proposed to mechanically stimulate embedded sensory receptors, resulting in pain and impaired movement [16,17]. Treatment consists of applying targeted manual pressure to the affected fascial tissue to mechanically correct proposed fascial distortions and restore normal fascial mechanics with the goal of reducing pain and improving function [16-18]. Although clinical studies have evaluated FDM across a variety of musculoskeletal conditions, the available literature remains heterogeneous with respect to study design, patient populations, comparator interventions, treatment protocols, and pain outcome measures. Existing reviews have primarily provided qualitative syntheses focused on individual musculoskeletal conditions, including chronic myofascial neck pain and adhesive capsulitis [19,20]. However, a quantitative synthesis of the available evidence evaluating FDM across musculoskeletal disorders has not been performed.

The primary objective of this systematic review and meta-analysis was to evaluate the effect of FDM on patient-reported pain outcomes measured by VAS or NPRS compared with alternative treatment approaches in individuals with musculoskeletal disorders. The secondary objective was to quantitatively evaluate within-group changes in pain following FDM using pooled pre- and post-intervention VAS or NPRS scores, irrespective of comparator, to further characterize the overall effect of FDM on musculoskeletal pain.

## Review

Materials and methods

Protocol 

This systematic review was conducted in accordance with the Preferred Reporting Items for Systematic Reviews and Meta-Analyses (PRISMA) 2020 guidelines, which guided study identification, screening, and selection [21]. Although this review was not prospectively registered, eligibility criteria, outcomes of interest, search strategies, study selection procedures, and planned analytical methods were established prior to study screening and data extraction.

Eligibility Criteria

The review question was structured according to the Population, Intervention, Comparator, Outcome, and Study Design (PICOS) framework. The population of interest included patients with musculoskeletal pathologies who received FDM treatment and for whom standardized pain outcome data were reported. The intervention of interest was FDM therapy, and the primary outcome was patient-reported pain measured using validated instruments, including the VAS and NRPS.

Studies were eligible if they included patients receiving at least one session of FDM for a musculoskeletal condition and reported standardized pre- and post-intervention pain outcomes. Randomized controlled trials, non-randomized interventional studies, cohort studies, and other clinical studies reporting human patient-level pain outcomes were eligible regardless of clinical setting, comparator group, or additional outcome measures. Studies evaluating multimodal interventions were included if FDM-specific outcome data could be extracted separately.

Review articles, systematic reviews, meta-analyses, conference abstracts lacking sufficient outcome data, animal studies, and studies evaluating manual therapy interventions other than FDM were excluded. Studies that did not report extractable pre- and post-intervention pain outcomes were also excluded. Gray literature lacking sufficient methodological or outcome data for quality assessment and quantitative synthesis was also excluded. This broad eligibility strategy was selected to comprehensively evaluate the association between the FDM and patient-reported pain across the spectrum of musculoskeletal conditions represented in the available literature.

Information Sources and Search Strategy

A comprehensive literature search was conducted to identify clinical studies evaluating the FDM. The databases PubMed/MEDLINE, Google Scholar, the Cochrane Library, Semantic Scholar, and ClinicalTrials.gov were searched from database inception through June 2026. Search strategies were adapted for each database to account for differences in indexing systems, controlled vocabulary, and search functionality while maintaining consistent conceptual domains related to the FDM. Search terms included "Fascial Distortion Model", "FDM", and related terminology describing fascial distortion treatment. All studies identified through the search strategy that referenced FDM were considered for screening. 

Study Selection and Data Extraction

**Figure 1:**
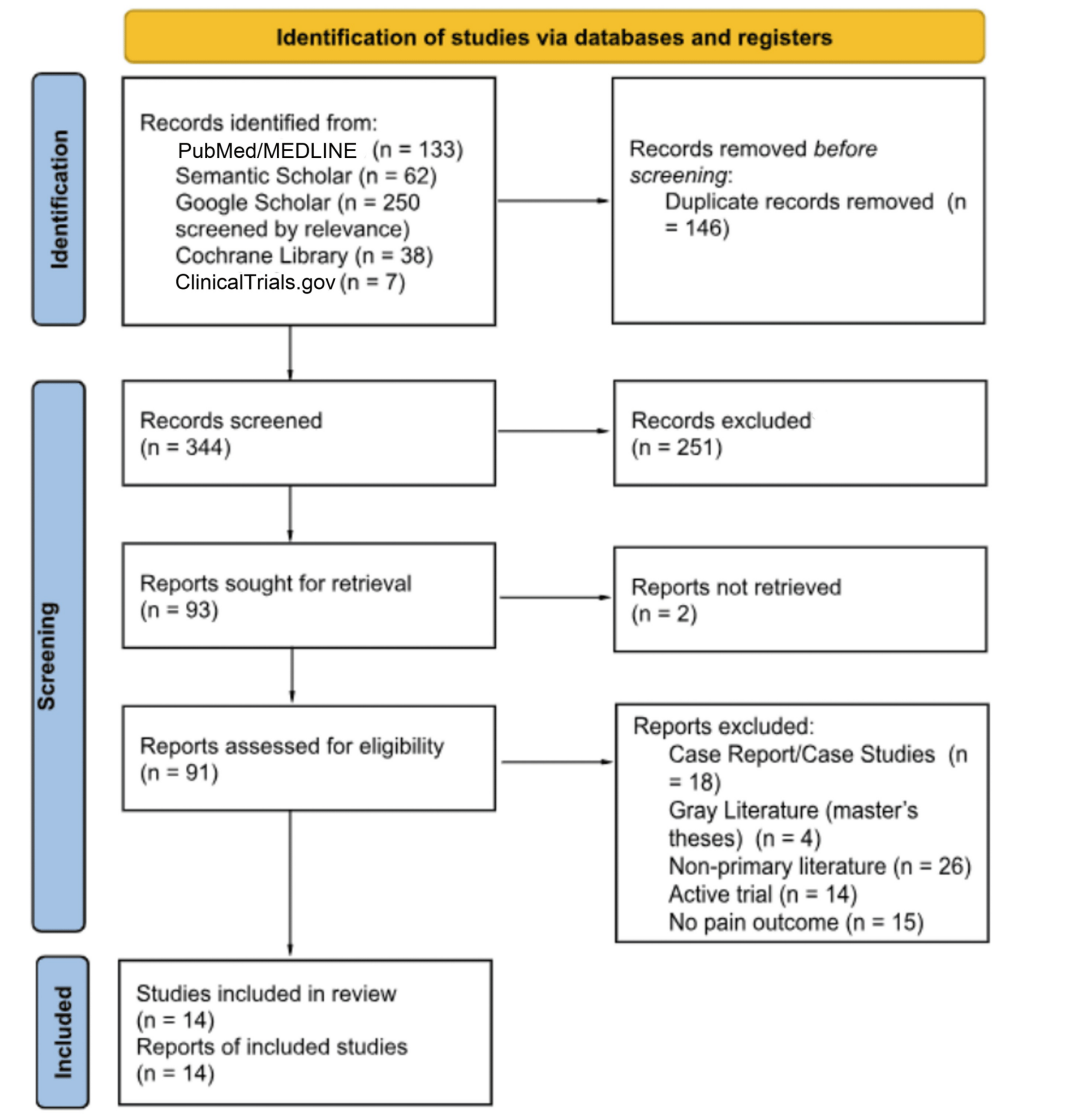
PRISMA flow diagram of the study selection process PRISMA: Preferred Reporting Items for Systematic reviews and Meta-Analyses

Literature searches, study selection, and data extraction were performed independently by two reviewers (A.P. and K.J.). Disagreements were resolved through discussion and consensus. Titles and abstracts were screened for relevance, followed by full-text review of potentially eligible studies. Reasons for exclusion at the full-text stage are detailed in Figure 1 [21].

Statistical Analysis

Pain outcomes reported using the NPRS or VAS were converted to a common 0-10 scale prior to analysis. When studies reported medians and ranges or interquartile ranges, means and standard deviations were estimated using established Cochrane-recommended conversion methods [22,23].

Two meta-analytic approaches were performed. The primary analysis compared post-intervention pain outcomes between FDM and non-FDM comparator interventions in randomized controlled trials. Mean differences (MDs) and corresponding 95% confidence intervals (CIs) were calculated using inverse-variance pooling under a random-effects model. 

Comparator interventions were not restricted to a single treatment modality, as the objective was to estimate the overall comparative effectiveness of FDM across clinically relevant alternative interventions. A random-effects model was selected prior to account for anticipated clinical heterogeneity among comparator interventions. This pairwise meta-analysis was conducted and visualized using NetMetaEasy (A5 Genetics Ltd, Kutaso, Hungary), a web-based meta-analysis platform [24]. 

The secondary analysis evaluated within-group pre-post changes in pain following FDM treatment across eligible studies. Mean change scores were calculated as post-treatment minus pre-treatment values, with negative values indicating pain reduction. Standard errors for change scores were estimated from reported pre- and post-treatment standard deviations assuming a pre-post correlation coefficient of 0.5. The pre-post meta-analysis was conducted using restricted maximum likelihood (REML) estimation in JASP (University of Amsterdam, Amsterdam, The Netherlands) [25].

For both analyses, pooled effect estimates and corresponding 95% CIs were calculated, and statistical significance was assessed using the test for overall effect. Between-study heterogeneity was evaluated using Cochran's Q statistic, the I² statistic, and between-study variance (τ²). Prediction intervals were calculated for the pre-post analyses to estimate the range of effects expected in future studies. Prespecified subgroup analyses of the pre-post meta-analysis included studies using diagnosis-specific FDM treatment approaches and randomized controlled trials only. Forest plots were generated for all primary and subgroup analyses. Publication bias for the primary comparative meta-analysis was planned to be assessed using funnel plots and Egger's regression test if at least 10 eligible studies were available, in accordance with Cochrane recommendations. These methods were not planned for the secondary pre-post analyses because they are intended for comparative treatment effect meta-analyses rather than pooled within-group analyses. All statistical analyses and calculations were independently verified by an independent biostatistician to ensure the accuracy of the reported results.

Risk of Bias Assessment

Risk of bias was assessed using study design-specific appraisal tools by two reviewers (A.P. and K.J.). The Cochrane Risk of Bias 2 (RoB 2) tool was used for randomized controlled trials [26], and the Risk Of Bias In Non-randomized Studies of Interventions (ROBINS-I) tool was used for non-randomized interventional studies [27]. Certainty of evidence on an outcome level was assessed using the Grading of Recommendations Assessment, Development, and Evaluation (GRADE) framework [28].

Results

Study Selection

The literature search and study selection process identified 14 studies that met the predefined eligibility criteria for inclusion in the qualitative and quantitative synthesis (Figure 1). Study screening, full-text eligibility assessment, and data extraction were performed independently by two reviewers (A.P. and K.J.), with disagreements resolved through discussion and consensus.

Study Characteristics

Characteristics of the 14 included studies are summarized in Table 1. A total of 644 participants were included across the studies, which evaluated FDM either as a standalone intervention or in comparison with manual therapy, guideline-based care, physical therapy, or other conservative interventions. Four studies assessed pain using the NPRS, whereas 10 studies used the VAS. In addition to pain outcomes, several studies evaluated secondary outcomes including range of motion and other functional measures. The included studies investigated a variety of musculoskeletal conditions involving the neck, trapezius, shoulder, tibia, heel, gluteal region, lower back, and calf.

**Table 1:** Summary of the included studies evaluating FDM on pain scale outcomes in patients with musculoskeletal pain This table summarizes the characteristics and pain-related findings of studies included in this systematic review evaluating the effects of the FDM on musculoskeletal pain. Information presented includes study design, sample size, body region of pain, FDM application approach, pain outcome measure, and the primary pain-related findings. Studies were identified through a comprehensive literature search of MEDLINE/PubMed, Google Scholar, Semantic Scholar, the Cochrane Library, and ClinicalTrials.gov. FDM: Fascial Distortion Model; RCT: randomized controlled trial; NPRS: Numeric Pain Rating Scale; VAS: Visual Analog Scale; MT: manual therapy; CMRT: Cellular Matrix Rhythm Therapy; NSAIDs: nonsteroidal anti-inflammatory drugs

Author	Study design	Population (n)	Body region of pain	FDM application	Pain scale	Key pain scale findings
Aroob et al. [29]	RCT	54	Gluteal	Protocol	NPRS	FDM showed a significant reduction in pain at week 6 compared to baseline. FDM with neuromuscular inhibition showed significantly greater reduction in pain compared to only FDM at week 6
Boucher et al. [30]	Single-arm prospective effectiveness study	28	Heel	Diagnosis specific	VAS	FDM showed a significant reduction in pain at one week and 16 weeks post-intervention compared to baseline
Dandgavhal and Metgud [31]	RCT	54	Trapezius	Protocol	VAS	FDM showed a significant reduction in pain post-intervention. FDM showed a significantly greater reduction in pain compared to Kinetic Chain Activation Technique post-intervention
Fink et al. [32]	RCT	60	Shoulder	Protocol based off diagnosis	VAS	FDM showed a significantly greater reduction in pain compared to conventional MT. FDM and MT showed a significant reduction in pain post-intervention. FDM experienced a greater amount of pain during treatment compared to MT
Ju [33]	RCT	30	Neck	Non-specific three-treatment protocol	VAS	FDM and foam rolling showed a significant reduction in pain post-intervention. There was no significant difference in pain reduction between FDM, foam rolling, and massage gun
Kaur et al. [34]	RCT	20	Neck	Diagnosis specific	NPRS	FDM + CMRT and FDM + sham both demonstrated statistically significant decreases in pain post-intervention. FDM + CMRT showed a significantly greater reduction in pain compared to FDM + sham
Kim and Lee [35]	RCT	45	Neck	Protocol	VAS	FDM showed a significant reduction in pain post-intervention. FDM showed a significantly greater reduction in pain at the six-week follow-up compared to myofascial release and self-myofascial release
Mannan et al. [36]	RCT	30	Calf	Two-treatment protocol	NPRS	FDM was associated with a significant reduction in pain. Foam rolling showed a greater improvement in pain compared to FDM at week 4
Moradi et al. [37]	RCT	30	Shoulder	Diagnosis specific	VAS	FDM showed a significantly greater reduction in pain than joint mobilization across all interventions and reduced pain faster across all treatments and two weeks post-intervention
Qureshi et al. [38]	RCT	44	Neck	Protocol	NPRS	FDM + isometrics and FDM without isometrics both showed a significant reduction in pain post-intervention. FDM + isometrics showed a significantly greater reduction in pain compared to FDM without isometrics
Richter et al. [39]	Prospective controlled trial	77	Lower back	Diagnosis specific	VAS	There was no statistically significant difference in pain reduction between the FDM and German National Disease Management Guideline groups. Between day 3 and week 2, the FDM group used significantly less analgesics and NSAIDs
Rogala et al. [40]	Prospective controlled clinical trial	50	Shoulder	Diagnosis specific	VAS	FDM showed a significantly greater reduction in pain compared to standard physiotherapy post-intervention. FDM showed a significant reduction in pain post-intervention
Schulze et al. [41]	Prospective single-arm controlled study	32	Tibia	Diagnosis specific	VAS	FDM showed a significant reduction in exercise-induced pain after one treatment and the final treatment
Wiaderna et al. [42]	RCT	90	Neck	Diagnosis specific	VAS	One session of FDM showed a significant reduction in pain

Risk of Bias

**Figure 2:**
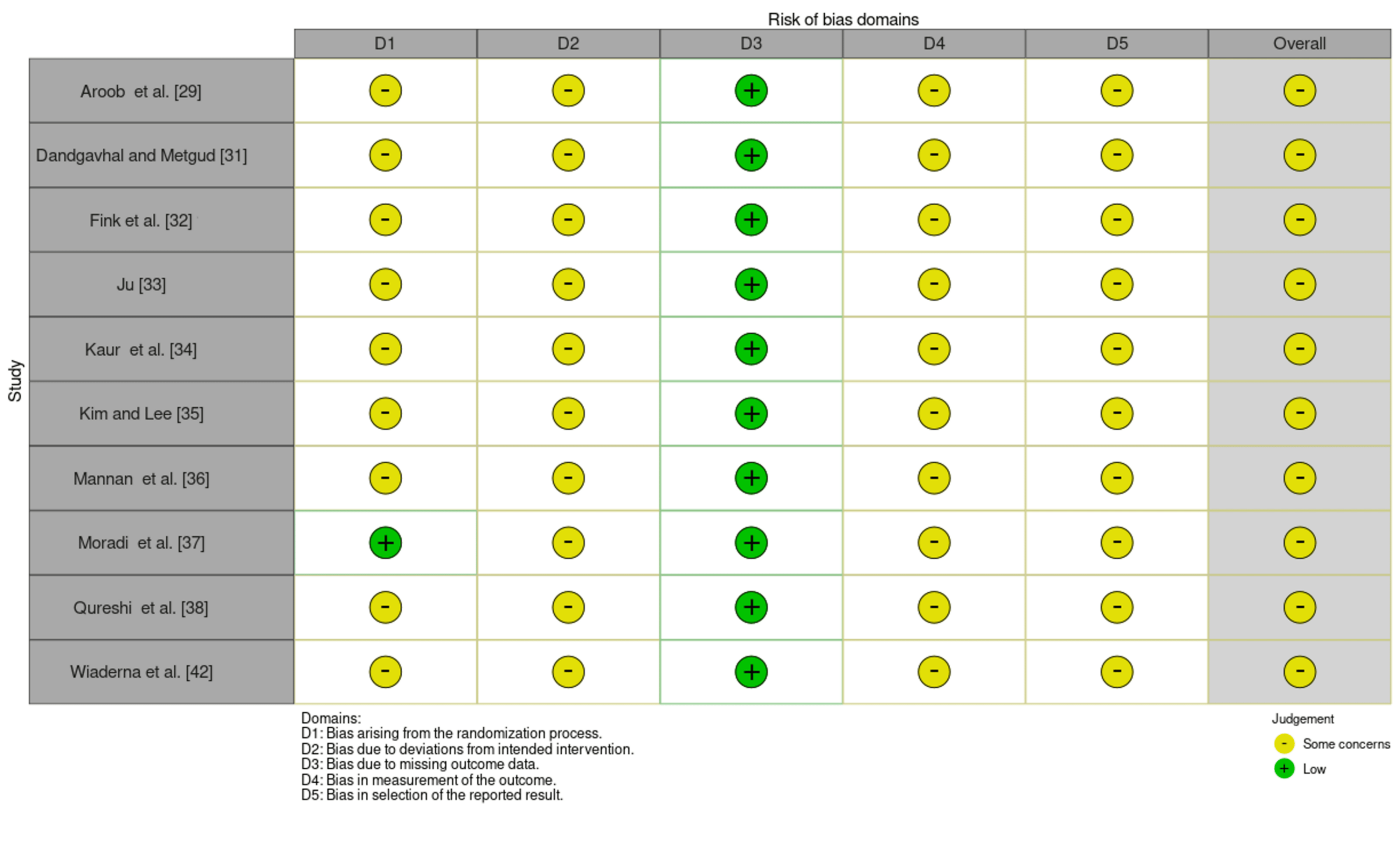
Risk of bias assessment of randomized controlled trials using the Cochrane Risk of Bias 2 (RoB 2) tool Risk of bias was evaluated across five domains: bias arising from the randomization process (D1), bias due to deviations from intended interventions (D2), bias due to missing outcome data (D3), bias in measurement of the outcome (D4), and bias in selection of the reported result (D5). Overall risk of bias judgments were assigned according to the RoB 2 algorithm.

Risk of bias among randomized controlled trials (n=10), assessed using the RoB 2 tool, was most commonly judged to raise some concerns, primarily arising from the randomization process and the selection of the reported result (Figure 2) [26].

**Figure 3:**
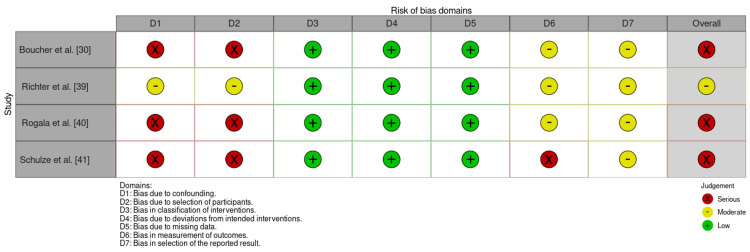
Risk of bias assessment of non-randomized interventional studies using the Risk Of Bias In Non-randomized Studies of Interventions (ROBINS-I) tool Risk of bias was evaluated across seven domains: bias due to confounding (D1), bias in selection of participants into the study (D2), bias in classification of interventions (D3), bias due to deviations from intended interventions (D4), bias due to missing data (D5), bias in measurement of outcomes (D6), and bias in selection of the reported result (D7). Overall risk of bias judgments were assigned according to ROBINS-I guidance.

Non-randomized interventional studies (n=4), evaluated using the ROBINS-I tool, showed overall moderate to serious risk of bias, largely due to confounding and participant selection (Figure 3) [27].

Using the GRADE framework, the overall certainty of evidence for pain outcomes ranged from very low to moderate, with downgrades primarily attributable to risk of bias and inconsistency across the studies (Table 2) [28].

**Table 2:** GRADE assessment of certainty of evidence for patient-reported pain outcomes Certainty of evidence was assessed using the GRADE framework for pain outcomes across all meta-analytic comparisons conducted in this review, including overall pre-post analyses, subgroup analyses, and comparative intervention analysis. Negative MD values indicate reductions in pain favoring FDM interventions. CIs represent the precision of pooled effect estimates. Prediction intervals estimate the range of effects expected in future studies and were included to facilitate the interpretation of between-study heterogeneity.  Risk of bias, inconsistency, indirectness, imprecision, and publication bias were assessed for each outcome according to GRADE methodology. FDM: Fascial Distortion Model; RCT: randomized controlled trial; GRADE: Grading of Recommendations Assessment, Development, and Evaluation; MD: mean difference; CI: confidence interval

Outcome analysis	Studies (n)	Effect estimate (95% CI)	Prediction interval	Risk of bias	Inconsistency	Indirectness	Imprecision	Publication bias	Overall certainty
FDM pre-post (all studies)	12	MD=-3.42 (-4.23 to -2.61)	-6.26 to -0.58	Serious	Serious	Not serious	Not serious	Undetected	Low
FDM diagnosis subgroup	5	MD=-4.14 (-5.86 to -2.42)	-8.28 to 0	Serious	Serious	Not serious	Not serious	Undetected	Very low
FDM pre-post (RCT only)	8	MD=-2.94 (-3.73 to -2.16)	-5.21 to -0.68	Not serious	Serious	Not serious	Not serious	Undetected	Moderate
FDM vs. comparator	6	MD=-0.92 (-1.70 to -0.14)	-3.46 to 1.63	Not serious	Serious	Not serious	Not serious	Undetected	Moderate

Study Outcomes

**Figure 4:**
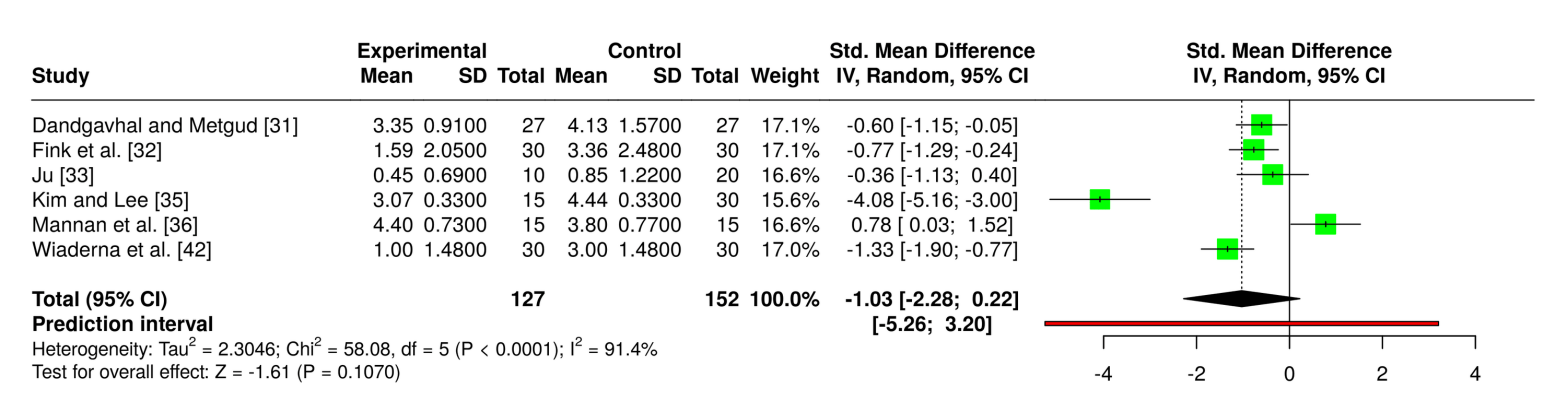
Forest plot of comparative analysis of FDM versus comparator interventions on pain outcomes Forest plot showing the pooled between-group effect of FDM versus comparator interventions on pain outcomes. Comparator interventions consisted of the control treatments used in each included study. Negative MD values favor FDM. Squares represent individual study effect estimates, with horizontal lines indicating 95% CI. The diamond represents the pooled effect estimate from the random-effects meta-analysis. FDM: Fascial Distortion Model; MD: mean difference; CI: confidence interval

FDM vs. comparative interventions: Seven studies compared FDM with alternative treatment approaches; however, one study was excluded from quantitative synthesis because it was not a randomized controlled trial. Consequently, six studies were included in the meta-analysis. Overall, the findings were favorable, with a pooled MD of -0.92 points (95% CI: -1.70 to -0.14; p=0.02) compared with alternative interventions (Figure 4).

Although excluded from the meta-analysis, Rogala et al. reported the largest between-group difference, with post-treatment pain scores of 0.56 in the FDM group compared with 6.71 in the comparator group (MD: -6.15) [40]. Among studies included in the quantitative synthesis, Wiaderna et al. observed the largest benefit favoring FDM (1.00 vs. 3.00; MD: -2.00) [42]. Fink et al. likewise reported lower post-treatment pain scores following FDM compared with the comparator intervention (1.59 vs. 3.36; MD: -1.77) [32]. Kim and Lee found lower pain scores in the FDM group than in controls (3.07 vs. 4.44; MD: -1.37) [35]. Dandgavhal and Metgud reported a smaller but favorable difference (3.35 vs. 4.13; MD: -0.78) [31]. Ju similarly observed lower post-treatment scores following FDM (0.45 vs. 0.85; MD: -0.40) [33]. In contrast, Mannan et al. reported slightly higher pain scores in the FDM group than in the comparator group (4.40 vs. 3.80; MD: +0.60) [36]. Collectively, five of the six studies included in the meta-analysis favored FDM over comparator interventions.

Further subgroup analysis restricted to randomized controlled trials comparing FDM with standard care or equivalent comparator interventions was not possible because of the limited number of eligible studies available for synthesis. The substantial heterogeneity observed across the studies likely reflects variation in comparator interventions, treatment protocols, and participant characteristics.

Pre-Post Pain Outcomes

**Figure 5:**
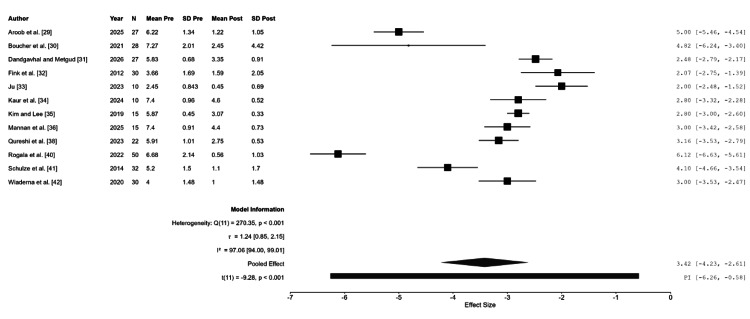
Forest plot of pre-post analysis of FDM interventions on pain outcomes Forest plot showing the pooled pre-post effect of FDM on pain outcomes across all included studies. Negative MD values indicate reductions in pain following FDM intervention. Squares represent individual study effect estimates, with horizontal lines indicating 95% CI. The diamond represents the pooled effect estimate from the random-effects meta-analysis. FDM: Fascial Distortion Model; MD: mean difference; CI: confidence interval

Fourteen studies evaluated the impact of FDM on pain outcomes, though two studies were excluded from the meta-analysis because results were presented only in graphical form and insufficient numerical data were provided for extraction. Without extractable quantitative values, these studies could not be incorporated into the quantitative synthesis. Overall, findings were favorable, with a pooled MD of -3.42 points (95% CI: -4.23 to -2.61) favoring FDM (Figure 5).

The two studies excluded from the quantitative synthesis also reported favorable outcomes following FDM treatment. Moradi et al. observed significantly greater pain reduction with FDM compared with joint mobilization in patients with shoulder impingement syndrome immediately following treatment, although between-group differences were no longer significant at the short-term follow-up [37]. Richter et al. similarly reported more rapid improvement in pain and reduced analgesic use with FDM compared with guideline-based management for acute non-specific low back pain, although long-term pain outcomes were comparable between groups [39].

Among the studies included in the quantitative synthesis, Rogala et al. demonstrated the largest improvement, with pain scores decreasing from 6.68 to 0.56 (mean change: -6.12) [40]. Aroob et al. reported a reduction from 6.22 to 1.22 (mean change: -5.00) [29]. Boucher et al. observed a decrease from 7.27 to 2.45 (mean change: -4.82) [30]. Schulze et al. found scores decreased from 5.20 to 1.10 (mean change: -4.10) [41]. Qureshi et al. reported a reduction from 5.91 to 2.75 (mean change: -3.16) [38]. Mannan et al. and Wiaderna et al. each observed decreases of 3.00 points, from 7.40 to 4.40 and from 4.00 to 1.00, respectively [36,42]. Kaur et al. and Kim and Lee both reported reductions of 2.80 points, with scores decreasing from 7.40 to 4.60 and from 5.87 to 3.07, respectively [34,35]. Dandgavhal and Metgud found scores decreased from 5.83 to 3.35 (mean change: -2.48) [31]. Fink et al. reported a reduction from 3.66 to 1.59 (mean change: -2.07) [32]. Ju observed the smallest improvement, with scores decreasing from 2.45 to 0.45 (mean change: -2.00) [33]. Collectively, all included studies demonstrated reductions in pain scores following FDM treatment.

**Figure 6:**
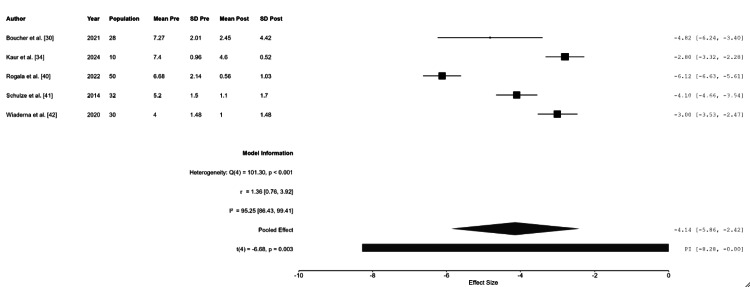
Forest plot of pre-post analysis of studies reporting specific FDM diagnoses Forest plot showing the pooled pre-post effect of FDM on pain outcomes among studies that reported specific FDM diagnoses. Negative MD values indicate reductions in pain following FDM intervention. Squares represent individual study effect estimates, with horizontal lines indicating 95% CI. The diamond represents the pooled effect estimate from the random-effects meta-analysis. FDM: Fascial Distortion Model; MD: mean difference; CI: confidence interval

**Figure 7:**
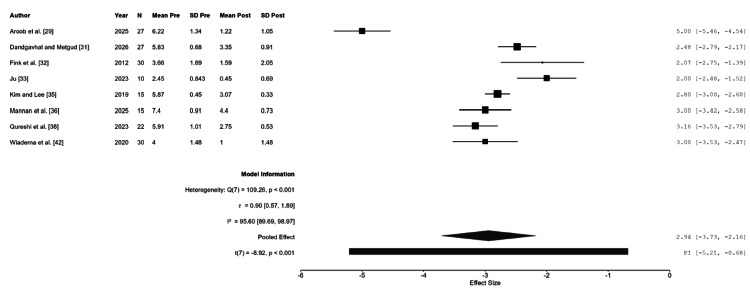
Forest plot of pre-post analysis of randomized controlled trials evaluating FDM interventions on pain outcomes Forest plot showing the pooled pre-post effect of FDM on pain outcomes among randomized controlled trials only. Negative MD values indicate reductions in pain following FDM intervention. Squares represent individual study effect estimates, with horizontal lines indicating 95% CI. The diamond represents the pooled effect estimate from the random-effects meta-analysis. FDM: Fascial Distortion Model; MD: mean difference; CI: confidence interval

Given the availability of sufficient data, subgroup analyses were performed for studies involving a specific FDM diagnosis (Figure 6) and for randomized controlled trials alone (Figure 7) to further explore the potential sources of heterogeneity and assess the robustness of the overall findings.

Discussion

FDM Compared to Other Interventions

The pooled analysis comparing FDM to alternative interventions demonstrated a statistically significant association favoring FDM (MD: -0.92; 95% CI: -1.70 to -0.14; p=0.021). Although the pooled effect favored FDM, the magnitude of the between-group difference did not reach a clinically meaningful threshold. Substantial heterogeneity was observed across the included studies (I^2^=91.2%), which indicated considerable variability in treatment effects between study populations and interventions. Furthermore, the prediction interval (-3.46 to 1.63) crossed zero, suggesting that future studies may demonstrate no meaningful difference when compared with alternative interventions in reducing pain. These findings should therefore be interpreted cautiously, as current evidence suggests that while FDM can reduce musculoskeletal pain, it does not appear to provide a clinically meaningful advantage over other treatment approaches.

FDM Effect on Pain

Overall, the pooled analysis comparing pain before and after FDM demonstrated a clinically and statistically significant association with pain reduction (MD: -3.42; 95% CI: -4.23 to -2.61; p<0.001), with substantial heterogeneity observed across included studies (I²=97.06%). Study results consistently trended in favor of FDM, although the magnitude of pain reduction varied between the studies. The mean prediction interval (-6.26 to -0.58) did not cross zero, suggesting that future studies would be expected to demonstrate a similar direction of effect despite the observed heterogeneity. These findings support a potential role of FDM in pain reduction; however, the substantial variability between studies limits confidence in the magnitude of the observed effect. 

A subgroup analysis was completed using studies that reported FDM diagnoses that demonstrated a larger associated pain reduction when compared with the main analysis (MD: -4.14; 95% CI: -5.86 to -2.42; p<0.001). Substantial heterogeneity was also noted in this subgroup analysis (I^2^=95.25%), and the prediction interval (-8.28 to 0) was notably wider than that observed in the primary analysis. While the pooled effect remains favorable, the broader prediction interval and persistent heterogeneity suggest greater uncertainty regarding the reproducibility of these findings across different patient populations and clinical settings. This uncertainty was further reflected in the GRADE assessment, which rated the certainty of evidence as very low, indicating that the true effect may differ substantially from the observed estimate.

An additional subgroup analysis was completed using only randomized controlled trials that demonstrated a smaller effect than the other aforementioned analyses (MD: -2.94; 95% CI: -3.73 to -2.16; p<0.001). Similar to the prior analyses, the heterogeneity was substantial (I^2^=95.60%); however, this subgroup demonstrated the narrowest prediction interval, which remained entirely below zero. This finding suggests a more consistent treatment effect among randomized studies and may provide the strongest support for a true association between FDM and pain reduction. In contrast to the very low certainty rating observed in the other subgroup, the certainty of evidence for the randomized controlled trial subgroup was rated as moderate. This difference suggests that, despite the smaller observed effect size, the estimate derived from randomized studies may represent a more reliable assessment of the true treatment effect. 

Summary

Comparisons with alternative interventions favored FDM; however, the between-group differences were not clinically meaningful, indicating that FDM may be similarly effective to other conservative treatment approaches rather than demonstrably superior. Overall, the findings of this review demonstrated that the FDM was consistently associated with clinically meaningful reductions in musculoskeletal pain across the included studies. Although substantial heterogeneity was observed, prediction intervals generally remained favorable toward pain reduction, suggesting a consistent direction of effect despite variability in the magnitude of treatment benefit. Collectively, these findings support FDM as a viable treatment option for musculoskeletal pain while highlighting the need for continued investigation to better define its comparative effectiveness. Ongoing registered clinical trials reflect continued interest in the FDM and may help address many of the limitations identified within the current body of evidence [43,44].

Limitations

Several limitations were identified within the included studies that affect the interpretation of these findings. Risk of bias was an important consideration, as several studies demonstrated serious methodological concerns but were retained in the quantitative analyses due to the limited number of available studies. Manual therapy interventions, including the FDM, are inherently difficult to blind, increasing the risk of performance and detection bias. Additionally, several studies had small sample sizes, limited allocation concealment, and inadequate reporting of randomization procedures, all of which increase the potential for biased effect estimates. Considerable heterogeneity was also observed in study design, patient populations, intervention protocols, and comparator treatments, with FDM techniques often being poorly described. Collectively, these methodological limitations reduce confidence in the precision and reproducibility of the reported treatment effects.

This systematic review is also subject to several limitations. Although multiple major databases and trial registries were utilized to maximize study identification, the literature search did not include Embase, Scopus, Web of Science, or the Cumulative Index to Nursing and Allied Health Literature (CINAHL). The omission of these sources may have resulted in missed relevant studies and could have affected the comprehensiveness of the evidence base. Although intentionally broad search strings (see Appendices) were used to maximize study identification, reliance on a limited number of search terms may have failed to identify studies that investigated the FDM using alternative terminology or that did not explicitly reference FDM in the title, abstract, or indexing. Variations in technique, intervention duration, and clinical diagnosis contributed to both clinical and methodological heterogeneity. An additional limitation was the clinical heterogeneity of the included populations. The included studies encompassed a variety of populations, including those without a specific medical diagnosis, as demonstrated by Mannan et al., in which the study population consisted of high-heeled shoe wearers [36]. Consequently, the pooled estimates should be interpreted with caution, as the magnitude of benefit associated with the FDM may vary across different patient populations. While the inclusion of a diverse cohort of patients with different causes of musculoskeletal pain improves the potential generalizability of these findings across clinical settings, it reduces the precision of effect estimates for individual pathologies.

As the primary quantitative synthesis included non-randomized studies, the pooled estimates reflect associations and should not be interpreted as evidence of a causal effect. The comparative meta-analysis was restricted to randomized controlled trials; therefore, Rogala et al. was excluded despite otherwise meeting the inclusion criteria [40]. Although this approach improved the methodological rigor of the comparative analysis, it reduced the available sample size and may have influenced the pooled estimates. The small number of studies included in each quantitative analysis also limited statistical power and increased the influence of individual studies on the pooled estimates. Additionally, the need to estimate the mean and standard deviation for Wiaderna et al. introduced additional variability and contributed to the observed heterogeneity, as reflected by the large calculated standard deviation [42]. This review was conducted in accordance with PRISMA guidelines but was not prospectively registered, which may limit its methodological transparency. An additional limitation was the inability to formally assess publication bias. Although the secondary pre-post analysis included more than 10 studies, funnel plots and Egger's regression test are not appropriate for pooled within-group pre-post analyses. Furthermore, the primary comparative analysis and remaining subgroup analyses included fewer than 10 studies, precluding a reliable assessment of publication bias. Consequently, the potential influence of publication bias on the pooled estimates cannot be excluded. Consequently, the overall certainty of evidence supporting these findings remains limited, and the reported associations should be interpreted with caution.

## Conclusions

Compared with other interventions, the FDM demonstrated favorable effects but did not appear to provide a clinically meaningful advantage. Nevertheless, FDM was consistently associated with clinically meaningful reductions in pain across the included studies. Although substantial heterogeneity was observed throughout the analyses, prediction intervals remained favorable toward pain reduction, supporting the consistency of the overall direction of effect. These findings should be interpreted cautiously given the considerable heterogeneity observed across the studies. Randomized controlled trials demonstrated a moderate-certainty association between FDM and reduced pain, providing support for a true treatment effect. Further adequately powered randomized controlled trials are needed to better define the magnitude of effect and comparative effectiveness of FDM relative to other treatments for musculoskeletal pain.

## Appendices

Search strings used for each respective database

The MEDLINE/PubMed search combined text words and Medical Subject Headings (MeSH) as follows: ( "fascial distortion model"[Title/Abstract] OR FDM[Title/Abstract] ) AND ( pain OR musculoskeletal OR rehabilitation OR manual therapy ).

The Cochrane Library search used ("fascial distortion model" OR "fascial distortion").

The Google Scholar query used ("fascial distortion model" OR (FDM AND fascial distortion)).

The Semantic Scholar search used ("fascial distortion model" OR (FDM AND fascial distortion)).

The search of ClinicalTrials.gov used “Fascial Distortion Model $FDM$” in the intervention/treatment slot.
